# Why is the presence of autoantibodies against GAD associated with a relatively slow progression to clinical diabetes?

**DOI:** 10.1007/s00125-020-05175-8

**Published:** 2020-05-26

**Authors:** Anette-Gabriele Ziegler, Ezio Bonifacio

**Affiliations:** 1 Institute of Diabetes Research, Helmholtz Zentrum München, German Research Center for Environmental Health, Munich-Neuherberg, Germany; 2 Forschergruppe Diabetes, Technical University Munich, at Klinikum rechts der Isar, Munich, Germany; 3 Center for Regenerative Therapies Dresden, Faculty of Medicine Carl Gustav Carus, Technische Universität Dresden, Fetcherstrasse 105, 01307 Dresden, Germany; 4 Paul Langerhans Institute Dresden of the Helmholtz Center Munich at University Hospital Carl Gustav Carus and Faculty of Medicine, Technische Universität, Dresden, Germany

*To the Editor* Type 1 diabetes is an autoimmune disease that can be diagnosed by the detection of islet autoantibodies at a pre-symptomatic stage. Autoantibodies associated with type 1 diabetes have been described against five autoantigens: insulin, GAD65, insulinoma-associated antigen-2 (IA-2), zinc transporter 8 (ZnT8) and tetraspanin 7 [1]. Multiple islet autoantibodies are found in the large majority of children who develop type 1 diabetes, whereas patients who develop type 1 diabetes in adulthood often present with only GAD antibodies (GADA). Moreover, patients without GADA are, on average, younger at diagnosis.

Recently, two studies report that the presence of GADA in children with multiple islet autoantibodies is associated with slow progression to clinical type 1 diabetes. The Bavarian Fr1da study tested over 90,000 children aged 2–5 years for pre-symptomatic type 1 diabetes (multiple islet autoantibodies) and identified 280 children with a pre-symptomatic disease, 235 of them with GADA [2]. Children with GADA had a significantly lower risk of developing the clinical stage of type 1 diabetes within a 3 year observation period than children with multiple autoantibodies without GADA (HR 0.43 [95% CI 0.25, 0.75]). The lower risk in the GADA positives was found for both the children with two or with three positive islet autoantibodies. A similar finding was obtained in the TrialNet Path to Prevention Study [3]. Among participants with two autoantibodies, those with GADA had less risk (HR 0.35 [95% CI 0.22, 0.57]) of type 1 diabetes than those without GADA. The results from these two studies raise the intriguing possibility that GADA may represent a reaction intended to protect the beta cell.

There are additional findings indirectly supporting the hypothesis that the formation of GADA may not be detrimental for the beta cell and may dampen the destructive immune response in type 1 diabetes. GADA alone pose a relatively low risk for type 1 diabetes. There are several GADA-positive diseases or conditions that do not progress to type 1 diabetes unless other diabetes-associated antibodies are present. In animal models, weekly injection of anti-GAD monoclonal antibody into NOD mice led to a delay in the onset of diabetes and a decrease in the severity of insulitis [4], and in humans, the transfer of GADA (or IA-2A) from mothers with type 1 diabetes to offspring provided relative protection against the development of multiple islet autoantibodies in childhood [5].

In considering potential scenarios in which GADA could be mounted to protect the beta cell, it may be helpful to recollect the mimicry between GAD65 and the PEVKEK-containing 2C protein of the Coxsackievirus B (CVB) 4. Several studies searched for cross-reactivity of autoreactive T cells and antibodies toward these PEVKEK peptides. Some evidence for mimicry was inconsistently observed, but the PEVKEK-containing region of GAD65 was not more frequently targeted in people with type 1 diabetes than healthy individuals, and the relevance of this sequence similarity is unresolved. We would like to speculate whether the GADA response could indeed be linked to beta cell CVB infection and may protect against disease progression. We have observed that antibodies that develop against CBVs are heterogeneous and do not always include a neutralising anti-VP1 component in young children [6]. Strikingly, the same study also found that there was a marked difference between the early CVB responses in the children who developed a GAD-dominated autoimmunity and those who developed an insulin-dominated autoimmunity. GAD autoimmunity was associated with the development of neutralising antibodies, whereas early insulin autoimmunity was associated with a neutralising antibody-deficient response. At the time we focused on the deficient response associated with the early insulin autoantibodies (IAAs), suggesting that these children may have been more susceptible to prolonged virus exposure, a scenario that seems consistent with recent stool virome data in the TEDDY (The Environmental Determinants of Diabetes in the Young) study [7]. However, a closer look at the GADA-associated competent response to CBV may be warranted.

Finally, the question also arises as to whether GADA may be used to delay the progression to type 1 diabetes and how could this be tested? A number of studies have investigated GAD vaccination in mice or people with pre-symptomatic or manifest type 1 diabetes. These trials had mixed success in delaying progression or maintaining residual beta cell function [8]. However, these trials were based on the underlying concept of inducing GAD tolerance and mainly included people who had GAD autoimmunity. No study has specifically given a GAD vaccine to children without GADA. A trial in IAA^+^/IA-2A^+^ children who have a particularly rapid diabetes progression [6, 7] may be an option to test whether the induction of GADA by vaccination could slow disease progression. Similarly, it may also be cautiously worth considering primary prevention with a GADA-inducing vaccine.

## Abbreviations

CVB: Coxsackievirus B
GADA: GAD antibodies
IAA: Insulin autoantibody
IA-2: Insulinoma-associated antigen-2
